# An all-ultrasound cranial imaging method to establish the relationship between cranial FUS incidence angle and transcranial attenuation in non-human primates in 3D

**DOI:** 10.1038/s41598-024-51623-5

**Published:** 2024-01-17

**Authors:** Aparna Singh, Sergio Jiménez-Gambín, Elisa E. Konofagou

**Affiliations:** 1 Department of Biomedical Engineering, Columbia University, New York, NY USA; 2 Department of Radiology, Columbia University, New York, NY USA

**Keywords:** Biomedical engineering, Medical research, Physics

## Abstract

Focused ultrasound (FUS) is a non-invasive and non-ionizing technique which deploys ultrasound waves to induce bio-effects. When paired with acoustically active particles such as microbubbles (MBs), it can open the blood brain barrier (BBB) to facilitate drug delivery otherwise inhibited due to the presence of BBB. One of the parameters that affects the FUS beam propagation is the beam incidence angle on the skull. Prior work by our group has shown that, as incidence angles deviate from 90°, FUS focal pressures attenuate and result in a smaller BBB opening volume. The incidence angles calculated in our prior studies were in 2D and used skull information from CT. The study presented herein develops methods to calculate incidence angle in 3D in non-human primate (NHP) skull fragments using harmonic ultrasound imaging without using ionizing radiation. Our results show that ultrasound harmonic imaging is capable of accurately depicting features such as sutures and eye-sockets of the skull. Furthermore, we were able to reproduce previously reported relationships between the incidence angle and FUS beam attenuation. We also show feasibility of performing ultrasound harmonic imaging in in-vivo non-human primates. The all-ultrasound method presented herein combined with our neuronavigation system stands to increase more widespread adoption of FUS and render it accessible by eliminating the need for CT cranial mapping.

## Introduction

In neurological diseases such as epilepsy, Parkinson’s, and Alzheimer’s, the first line of treatment is typically pharmacological. These medications, however, have not been shown efficacious over time due to developed tolerance^1^ and progression of disease. In those cases, brain stimulation methodologies, such as deep brain stimulation (DBS) can treat the symptoms especially in advanced stages of Parkinson’s disease where in one randomized trial, DBS of subthalamic nucleus caused greater improvements from baseline to six months in quality of life in Parkinson’s disease patients when compared to medication alone^1^. However, DBS has resulted in serious adverse events in 13 percent of the cases including a fatal intracerebral hemorrhage, possibly due to it being invasive in nature^2^. Another widely used brain stimulation method, transcranial magnetic stimulation (TMS), has shown reduction in seizures in patients with epilepsy with longer stimulation groups reporting fewer seizures than shorter stimulation groups^3^. However, TMS suffers from poor depth penetration and limited spatial resolution^4,5^.

Focused ultrasound (FUS) is an exciting, relatively new alternative technique as it is not only inherently non-invasive, but also achieves greater depth penetration. Several clinical trials have shown the potential of FUS for treating essential tremors^6,7^, for providing pain relief^8^ and, for blood–brain barrier (BBB) opening applications^9,10^. For all FUS-guided therapies, the gold standard method for targeting is MRI^6,7,9,11^. MRI provides excellent tissue contrast with tissue temperature monitoring capabilities^12^ and helps with predicting therapeutic outcomes such as FUS ablation. However, it fails to monitor microbubble activity, which is paramount to safe and successful FUS BBB opening procedures. A second imaging modality that is widely used for FUS pre-planning is CT as it provides with important acoustic parameters of the skull and brain that are essential to predicting FUS beam path, attenuation of FUS pressures due to thickness and density of skull and predicting the incidence angle of FUS beams onto the skull^13–15^. However, CT is costly, ionizing, and without intra-monitoring capabilities.

Another alternative for guidance and particle activity monitoring is ultrasound. An ultrasound imaging array, when set on receive mode, can monitor this microbubble activity at high frame rates and inform us of safety of neuromodulation procedures or blood–brain barrier (BBB) opening. Past research published by our group has developed methods to detect microbubble activity during BBB opening^16,17^. Other significant developments have been reported over the past few years where ultrasound imaging arrays have been used to identify anatomical sutures on mice skull and has been used to guide blood–brain barrier opening in small animals and deliver molecules across the BBB^18,19^. Additionally, advancements in B-Mode^13^ image processing have made it possible for ultrasound imaging transducers to be used for brain vascular imaging^20,21^, perform transcranial imaging through the human skull^22^, and for detecting functional activity in brain in small animals^23,24^ and in newborns^25^. Overall, these recent developments of ultrasound-guided focused ultrasound technologies enabled the use of transcranial power Doppler image to guide BBB opening in rats^26^. Ultrasound monitoring has also played a huge role in other neuromodulation procedures that do not involve BBB opening. In one study by our group^27,28^, researchers showed that during neuromodulation of peripheral nervous system (PNS), ultrasound imaging transducer can be used to image real time displacement and cavitation, thereby informing us about the intricate interplay of cavitation and displacement in causing neuromodulation of PNS. In addition, other groups have also shown that ultrasound can be used to detect drug release from nanoparticles after FUS application^29^. Thus, ultrasound imaging guidance and monitoring can provide an efficient, reliable and promising alternative for FUS applications. In this study, we aimed to further explore the capability of real-time ultrasound harmonic B-Mode images for FUS applications. Studies published by our group has shown that incidence angle, angle between the normal vector to skull plane and the transducer plane, is critical for the reliable and reproducible BBB opening in non-human primates^13^ and humans^30–32^. In this study, we developed methods to use an all-ultrasound technique which can perform cranial imaging and predict FUS incidence angles on the skull using ultrasound imaging. We validated this technique in two ex-vivo NHP skulls and showed the feasibility of transcranial imaging in an in-vivo NHP study.

## Results

### k-Wave simulation predictions

**Figure 1:**
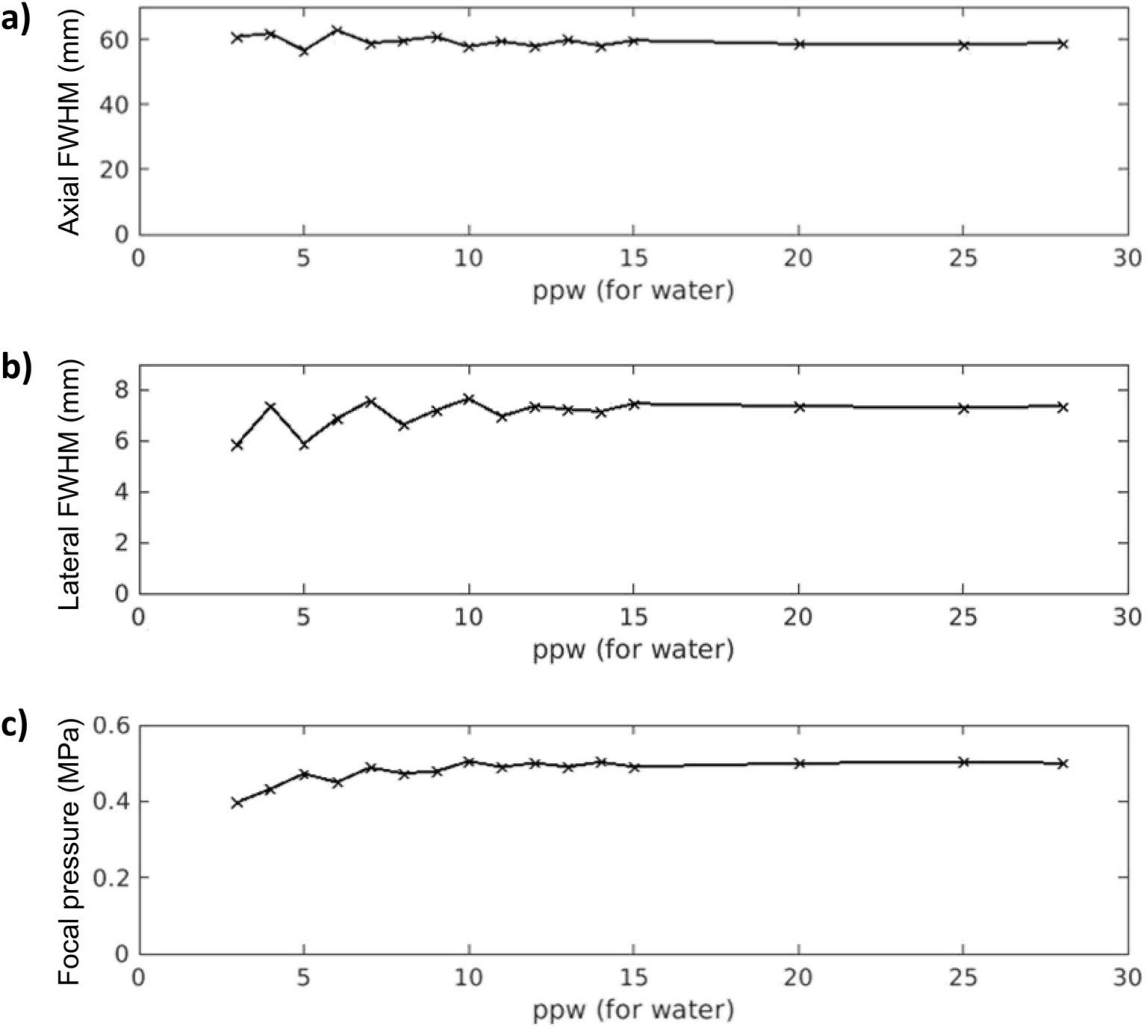
Simulation convergence assessment by evaluation of the focal properties of the single-element transducer modeled in free-field (i.e., water) at grid resolutions ranging from 3 ppw to 28 ppw. (**a**) Axial full width at half maximum. (**b**) Lateral full width at half maximum. (**c**) Focal peak pressure. Note that the range of 3-28 ppw in water corresponds to 8-74 ppw for skull tissue since its maximum sound speed is 2.66 times that of water.

Before performing the definitive k-Wave simulations, we evaluated the simulated grid resolution influence on the resulting focal properties of the single-element transducer in free-field. We performed a set of simulations of the FUS transducer increasing the particles per wavelength (ppw) in water from 3-28 (8-74 for skull), comparing the full width at half maximum (FWHM) in the axial and lateral directions, as well as the focal maximum pressure value, as shown in Fig. 1. At around 10 ppw (27 for skull), the axial and lateral FWHM are around 59 mm and 7 mm, respectively, and the focal pressure reaches 0.5 MPa. Lower ppw can still provide an accurate value as this transducer has a large focal spot size (59 × 7 mm), so that an error of 12 mm in the axial direction would not be relevant. Note that the simulation performed at 28 ppw (last result plotted on the right of each graph in Fig. 1) was the most complex, in terms of computational cost, that our GPU-based computer could carry out. Then, we set 15 ppw (as described in “Materials and Methods” section) as the grid resolution for all the simulations performed in this study. Then, we positioned the skull in MATLAB with different orientations, and we calculated the incidence angle by computing the angle between the normal vector fitted on to the skull plane and the normal vector corresponding to the transducer plane (Fig. 3c).

**Figure 2:**
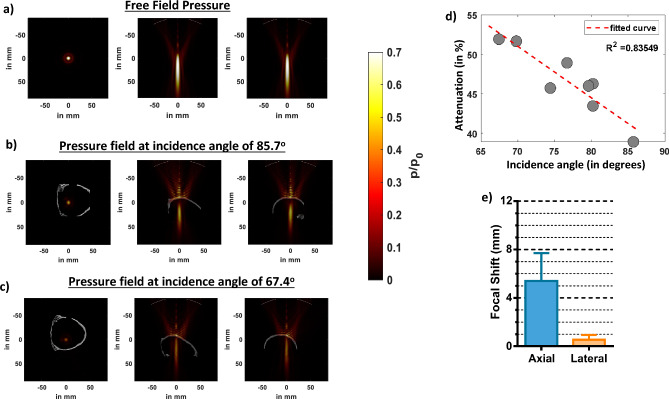
Effects of different FUS incidence angles on ex-vivo skull predicted by k-Wave simulations. (**a**) Lateral and axial pressure fields distributions, respectively, in free field propagations. (**b**) Lateral and axial pressure fields distributions in presence of skull at the best incidence angle of 85.7 degree. (**c**) Lateral and axial pressure fields distributions at an incidence angle of 67.4 degree. (**d**) Average axial and lateral focal shift computed from pressure fields in presence of skull at all incidence angles. (**e**) Graph showing a trend of increased attenuation away from normal incidence angle (90 degrees).

The results of k-Wave simulations (Fig. 2) show that the presence of skull at different incidence angles attenuate pressure fields where the pressure attenuated by as much as 47% when the incidence was 85.7 ° in Fig. 2b. The attenuation further increased to up to 60 % when incidence angle was 67.4 ° in Fig. 2c. We then compared how attenuation was affected at different FUS incidence angles to the skull in Fig. 2d. We observed that as the incidence angle approximated to 90 degrees, the attenuation decreased and that there was a strong correlation (R^2^ = 0.84) between the attenuation and FUS incidence angle. We then used all our eight simulations and compared the focal shift with respect to the free field in Fig. 2e and found that the average axial and lateral focal shift was 5.0 ± 2.4 mm and 0.38 ± 0.27 mm respectively.

### 3-D reconstruction of the cranial NHP skull map

**Figure 3:**
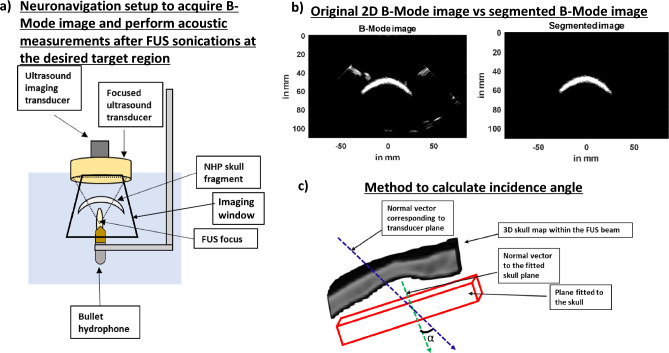
Transcranial skull imaging using ultrasound imaging transducer P4-2. (**a**) NHP skull fragment B-Mode images were acquired using P4-2 by performing raster scan of the entire skull. (**b**) B-Mode image (left) was segmented (right) below to only contain the skull. (**c**) Angle between normal vector fitted to the skull plane and normal vector corresponding to transducer plane was used to calculate incidence angle.

To generate a skull map, we manually segmented the skull from each of the 500 reconstructed, harmonic B-Mode images with a resolution of 0.14 × 0.14 × 0.2 mm (Fig. 3b). We then assembled each of those planes to generate a skull mask using the volshow function of MATLAB.

**Figure 4:**
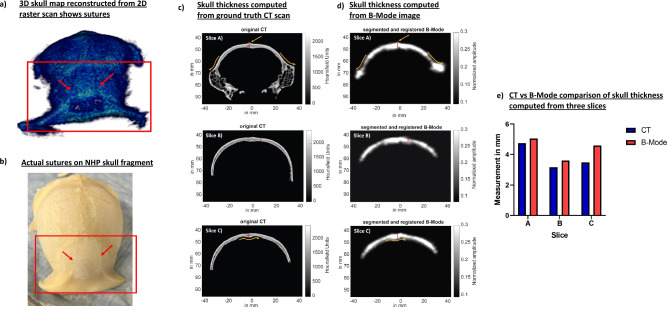
(**a**) 2D raster scans of skull #1 helped reconstruct 3D skull map. This 3D skull map shows sutures. (**b**) The sutures visible on 3D skull map were also present on the physical skull. (**c**) Skull thickness was computed using CT of the skull. Red line shows the region that was measured. (**d**) B-Mode image of the skull consisted of the same anatomical region. Red line denotes the region where measurements were computed from. (**e**) Skull thickness was computed from 3 different CT and B-Mode slices where anatomical regions could be matched. Comparing skull thickness values evaluated from CT and B-Mode show that the values between them were comparable.

The 3D skull map reconstructed from 2D raster scans for skull number 1 in Fig. 4a shows the skull sutures identified using the red arrows. These sutures are also present in the physical skull in Fig. 4b. We then performed rigid registration of the B-Mode map to the CT map. Then, we compared corresponding slices in CT (Fig. 4c) and reconstructed harmonic B-Mode (Fig. 4d), observing similar features (in yellow arrows) present in both CT and B-Mode slices. Furthermore, we overlaid B-Mode volume on CT volume in Supplementary Fig. 1 to show that the features and overall geometry register accurately. Thereafter, we calculated the thickness of the bone in those slices. We compared bone thickness in a total of 3 slices in Fig. 4e and found that the thickness computation from B-Mode and CT slices were comparable (within 1 mm error). In slice A, the measurements computed from CT vs B-Mode were equal to 4.75 mm vs. 5.06 mm, in slice B it was 3.16 mm vs. 3.60 mm, and in slice C it was 3.48 mm vs. 4.60 mm. In addition, the average thickness across a circular skull region of 15 mm in radius, with the center at 0 mm in the horizontal axis (see in Fig. 4c,d for horizontal axis reference), was calculated for both CT and segmented-registered B-Mode, resulting in 3.37 mm and 4.41 mm, respectively. The skull map volume within this circular region was also computed, where the CT provided 2339 mm^3^ compared to the 3058 mm^3^ by the B-Mode.

**Figure 5:**
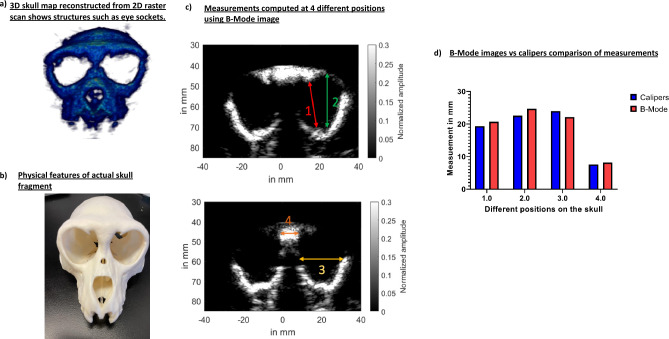
(**a**) 2D raster scan of skull #2 helped reconstruct its 3D skull map. We can see the eye sockets along with other prominent structures. (**b**) Physical skull also shows prominent features present on the 3D skull map. (**c**) A total of 4 measurements were taken to compute dimensions of eye sockets and its neighboring areas using its B-Mode image. (**d**) The B-Mode computed measurements were then compared to physical caliper measurements. These measurements were found to be comparable.

The 3D skull map for NHP skull # 2 in Fig. 5a clearly shows eye sockets and features that are present in the orthogonal skull in Fig. 5b. We compared the physical measurements of the eye sockets computed via calipers (Mitutoyo, Aurora, IL) with that of computed from B-Mode at 4 different positions shown via different color arrows in Fig. 5c. We found that the measurements, of calipers vs. B-Mode (see Fig. 5d), for location 1 was 19.34 mm vs. 20.7 mm, for location 2 was 22.57 mm vs. 24.7 mm, for location 3 was 24 mm vs. 22.17 mm, and for location 4 was 7.6 mm vs. 8.17 mm.

### Ex-vivo incidence angle estimation using harmonic B-Mode imaging and FUS pressure attenuation

**Figure 6:**
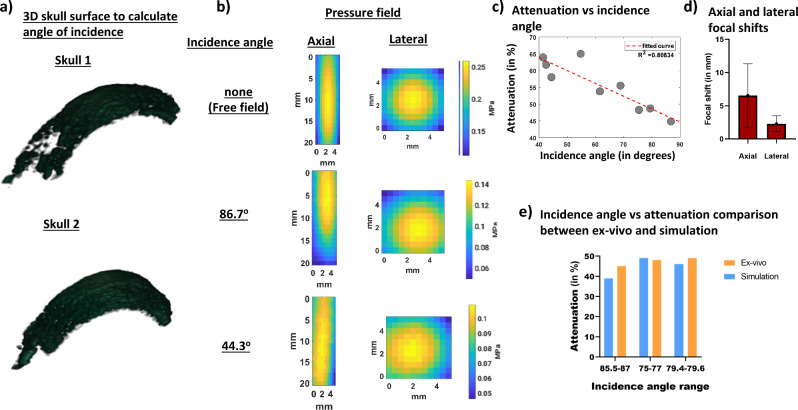
(**a**) Example of a 3D surface of the skull that was used to calculate incidence angle for both skulls. A total of 9 3D surfaces were used to calculate incidence and angle and establish its relationship with attenuation. (**b**) Pressure fields recorded in free field and transcranially at the best and worst incidence angle show a reduction in attenuation as incidence angle gets further away from 900. (**c**) Graph that shows relationship between incidence angles, calculated from 3D B-Mode images, and attenuation calculated from pressure fields. (**d**) Focal shifts evaluated from pressure fieldmaps recorded using hydrophone at different incidence angles. (**e**) Graph comparing similar incidence angles between ex-vivo and simulations.

To calculate the incidence angle through two ex-vivo NHP skulls, we acquired and segmented 70 slices of harmonic B-Mode images, each 0.5 mm apart using the imaging transducer that was co-axially aligned with our FUS transducer. Our imaging sequence comprised 256 diverging waves acquired at 2-MHz with phased array (P4-2, ATL, Philips). B-Mode images were acquired at a depth of 110 mm. To obtain a single harmonic B-Mode image, a 2-cycle diverging wave at 2 MHz followed by another 2-cycle diverging wave with opposite polarity was delivered. The final reconstructed B-Mode image was then saved onto the computer and the transducer was then moved to the next plane 0.5 mm away to ultimately acquire 70 slices. We used this partial 3D skull map in Fig. 6a for both skulls to estimate FUS incidence angle on the skull (Fig. 3c). We then performed FUS sonications with our H-231 FUS single-element transducer (Sonic Concepts, Bothell, WA). Our FUS transducer (F0 = 250 kHz, OD = 110 mm, ID = 44 mm, and focal distance = 110 mm), was coaxially aligned with the imaging transducer. FUS pulses of 15 cycles at a PRF of 100 Hz at 0.3 MPa were transmitted thereafter and we used bullet hydrophone (H0400, ONDA Corporation, Sunnyvale, CA) to record field pressures underneath the skull. For skull #1 in Fig. 6b, we observe that the FUS incidence angle on the skull impacts the intensity of transcranial FUS pressure field recorded via hydrophone. As a result, similar to what was predicted in the simulation an incidence angle of 86.7 degrees shows lower attenuation than incidence angle of 44.3 degrees. When attenuation results are combined for both skulls in Fig. 6c, the same linear relationship as that of the simulation is obtained whereas the incidence angle approached 90 degrees, the attenuation decreased. The dependence of attenuation on the incidence angle was found to be high (R^2^ = 0.81). We also calculated average axial and lateral focal shifts in Fig. 6d from the beam path generated and found an average axial focal shift of 6.54 ± 4.81 mm and lateral focal shift of 2.31 ± 1.19 mm. When examining incidence angles vs attenuation, there were 3 incidence angles in Fig. 6e that were comparable between simulation and experimental condition. An incidence angle of 85.7 degrees in simulation resulted in 39 % attenuation whereas an incidence of 86.7 degrees in experimental condition resulted in 45 % attenuation. Similarly, an incidence angle of 76.7 degrees in simulation resulted in 49 % attenuation whereas an incidence of 75.4 degrees in experimental condition resulted in 48 % attenuation. Finally, an incidence angle of 79.6 degrees in simulation resulted in 46 % attenuation whereas an incidence of 79.4 degrees in experimental condition resulted in 49 % attenuation.

### In-vivo cranial harmonic B-mode imaging

**Figure 7:**
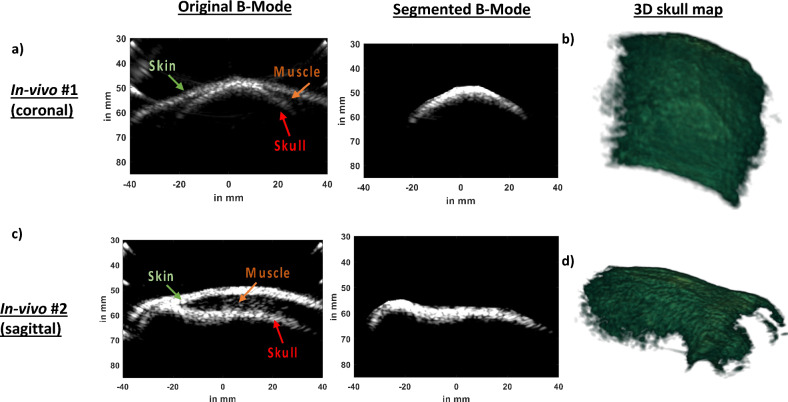
(**a**) A coronal slice ofIn-vivo B-Mode scan shows the skin, muscle, and skull. We took multiple B-Mode coronal slices of our first in-vivo NHP and segmented the skull from each of those B-Mode images (**b**) We reconstructed a 3D skull map using multiple coronal slices which shows sutures visible on the skull surface. (**c**) A sagittal slice of our second in-vivo study also reveals the skin, muscle, and skull. We segmented the skull from the B-Mode image. (**d**) After segmenting the skull out from multiple sagittal slices of our second in-vivo study, we reconstructed 3D skull map which shows eye sockets.

We performed cranial B-Mode harmonic imaging in two in-vivo NHPs (Rhesus Macaques, Male, 8 years old) using our ultrasound guided FUS setup driven by Robotic Arm (Universal Robots, UR5E). In our first NHP imaging study, we collected coronal slices. In the original B-Mode image in Fig. 7a, we can identify skin, muscle, and skull. We manually segmented out the skull from original B-Mode image (Fig. 7a) and created 3D skull map in Fig. 7b. In the 3D skull map rendered using the ‘volshow’ function of MATLAB, sutures inside the skull surface are depicted which is a characteristic of NHP skull. In our second in-vivo setting in Fig. 7c, we performed imaging in the sagittal plane. Similar to our first in-vivo imaging case, we identified skin, muscle, and skull layers. An observer manually segmented the skull based on the distinct intensity and assembled all 500 slices to generate a 3D skull map in Fig. 7d. In the sagittal plane, we were able to recover eye sockets.

## Discussion

FUS is a non-invasive and non-ionizing therapeutic technology that can treat neurological conditions by focusing ultrasound waves at desired target regions. Due to its non-invasive nature, it has been FDA approved for ablation for uterine fibroids and essential tremors. The clinical method for targeting FUS is using MRI thermometry^7,33,34^. To target accurately and calculate FUS attenuation for transcranial applications, CT images of the skull are utilized^35,36^. Such targeting methods have been used towards blood brain barrier opening and neuromodulation procedures. However, using MRI and CT collectively for targeting and trajectory planning can render FUS ionizing and less accessible. In this study, we demonstrated the feasibility of using transcranial ultrasound B-Mode imaging to compute the incidence angle during FUS for BBB opening in NHP using a clinical system^37^. Using an all-ultrasound system is advantageous when compared to other modalities such as MRI and CT. In addition to providing real-time guidance, ultrasound also provides tools for real-time monitoring thereby, enabling performing repeated FUS procedures reliably and cost-efficiently. Our neuronavigation clinical system, which has been used in this study, has successfully performed BBB opening and by developing methods to image skulls and accurately predict FUS incidence angles, we can enhance the applicability of our clinical system for FUS procedures. We showed that we can successfully use this clinical system to achieve our desired objective and that our experimental results are comparable to simulations. Our simulation results showed a determinant coefficient of 0.85 between FUS angle incidence and attenuation. This is in line with published studies where a similar relationship and determinant coefficient was seen between volume of BBB opening and incidence angle at fixed input pressures^13,14^.

### Cranial harmonic images of the skull accurately depicted skull landmarks

Cranial B-Mode harmonic images of ex-vivo skulls in Fig. 5 were shown capable of delineating skull boundaries. This enabled segmentation of the skull and eventually helped with 3D skull map reconstruction. The 3D skull map was able to show sutures and eye sockets present on the skull. Furthermore, B-Mode images were able correctly infer the skull thickness and eye socket width measurements. These will be unprecedentedly advantageous for targeting purposes as these anatomical sutures and locations can be further used with our neuronavigation system for real-time registration and FUS guidance^30,31,37–39^.

### Comparison between simulation and experimental findings

Our ex-vivo pressure measurements were found to be in good agreement with simulations. A determinant coefficient of 0.81 was observed in experimental conditions which was in agreement with our simulations. Ex-vivo pressure attenuation measurements closely resembled simulation pressure attenuation. When comparing attenuation values between simulation and physical measurements, the average error was within 6 %. Furthermore, the average axial focal shifts were similar in magnitude when experimental conditions were compared to simulations. However, the lateral focal shift was found to be 2 mm greater in experiments than in simulations. The average lateral shift in both cases was calculated by averaging contributions from all incidence angles. In experimental conditions, some of the incidence angles were lower than 45 degrees. On the other hand, in the simulations, lowest incidence angle was 69.8 degrees. A wider of incidence angle may have, thus, contributed to larger average lateral shift the in experimental condition.

### Invivo harmonic imaging revealed certain features on the NHP skull

We further showed feasibility of acquiring harmonic B-Mode images in two different in-vivo cases. In both cases in Fig. 6, the skin, muscle, and skull layers could be clearly distinguished. Furthermore, after segmentation and reconstruction of skull map, sutures and key anatomical landmarks such as eye sockets were clearly delineated. These features will enable us to use harmonic B-Mode imaging derived landmarks to guide and open the BBB using our clinical system in NHP.

Overall, the clinical impact of this work includes using real-time ultrasound imaging to guide the FUS procedures. This would enable a complete ultrasound guided FUS during neuronavigation procedures, in addition to removing the need for using the ionizing CT for transcranial FUS propagation simulation predictions. While our method can perform cranial harmonic B-Mode imaging and predict the angle of incidence in transcranial FUS applications, it has some limitations. This method relies on slice-by-slice segmentation which can present some challenges. Manual segmentation can be time consuming and subjective and need to be done off-line. For the next steps, we envision that manual segmentation paves the path for automated segmentation. Ongoing efforts to obtain the automated segmentation include implementing a machine learning algorithm which will then be built into the neuronavigation procedure to allow for online skull segmentation.

## Conclusion

In this study, we developed cranial harmonic B-Mode imaging method to predict angle of incidence for FUS therapies, thereby eliminating the need to use ionizing methods, such as CT, for targeting purposes. We, first, performed cranial imaging employing a clinical neuronavigation system, with ultrasound imaging transducer coaxially aligned with FUS transducer, on ex-vivo skulls. We used these cranial images to predict FUS beam incidence angle on the skull and compared it FUS pressure field recorded, via bullet hydrophone, at those respective incidence angles. The results showed a decreasing trend in attenuation as incidence angle approximated to normal. This agreed with our k-Wave simulation results and previously published results^13,14^. We, then, showed feasibility of performing harmonic cranial imaging in in-vivo NHPs. Our future work will incorporate in-vivo harmonic cranial imaging for targeting blood brain-barrier opening in large animals and clinical studies and will be used to calculate FUS incidence angles during brain therapies in real-time.

## Materials and methods

### Simulations using k-Wave

Numerical simulations to predict focused ultrasound pressure through NHP skull were modeled using k-Wave package^40,41^. This method is selected as it provides low numerical dispersion as compared with finite-differences methods^42,43^. We used the GPU-optimized package from k-Wave and we employed the 3-D acoustic simulations on an NVIDIA DGX Station (Nvidia, Santa Clara, CA).

First, an ex-vivo NHP skull was degassed for 24 h. Then, a CT scan of the skull fragment was acquired using a clinical CT scanner (Siemens BioGraph mCT 64 Slices Scanner, Siemens Healthcare), with a resolution of 0.24 × 0.24 × 0.6 mm^3^. The CT data was converted from Hounsfield Units to heterogeneous acoustic properties of sound speed and density using the linear-piecewise polynomials proposed previously^44,45^.

The absorption in k-Wave was modeled by following the power law expression α = α_0_f^y^, where α is the absorption value in dB/cm, α_0_ is the absorption coefficient in dB/cm/MHz^y^, f is the working frequency in MHz, and y is the power law exponent. The absorption map of the NHP skull was modeled as heterogeneous^46^, where the maximum absorption was calculated from the literature^44^ as α = α_0_f^y^ = 2.7(0.25^1^) = 0.68 dB/cm at our working frequency of 0.25 MHz. Afterwards, considering that the k-space method does not allow a spatial variation of the power law exponent^47^, it does not accept the unity for its definition, and we were working at a single FUS frequency, we chose the value y’ = 2 (water tissue) for the power law exponent, and then calculated the corresponding absorption coefficient α_0,new_ (referred as alpha_coeff in k-Wave) that provides the correct skull absorption of 0.68 dB/cm (α_0,new_ = α/f^y’^ = 0.68/0.25^2^ = 10.8 dB/cm/MHz^2^). Note that the absorption value internally calculated by k-Wave is α = α_0,new_f^y’^ = 10.8(0.25^2^) = 0.68 dB/cm. In this way, we can include as many different tissues as we need in our single-frequency simulation by easily adjusting its absorption coefficient α_0_.

Regarding the simulated grid size, the simulations reported in this study correspond to a single-frequency transducer (i.e., 250 kHz) for the experimental FUS procedure, therefore, higher frequencies (smaller wavelengths) corresponding to harmonics do not play a role for the focused beam propagation through the skull, which leaves the simulated grid size dependent on a single frequency only. Note that previous studies of single-frequency FUS propagation in free-field, through mouse, non-human primate and human skulls showed accuracy within 4–6 ppw for k-space^13,42,48,49^ and around 10 ppw for FDTD^35,43^. All of these spatial resolutions correspond to the smallest and therefore the limiting wavelength in the simulated media, which is water. For harder tissues, such as the skull, which have a higher sound speed and consequently a larger wavelength (2.66 times that of water in our simulated configuration), the relative grid size of 4-10 ppw for water corresponds to 10-25 ppw, and therefore the simulation accuracy is higher. In our case, we used an isotropic grid with a spatial step of 0.4 mm, which corresponds to 15 ppw in free-field (38 ppw for skull) at the working frequency. These 15 ppw corresponded to a minimum of 8 grid points across the NHP skull thickness, allowing to capture with more detail the microstructure and irregularities of the skull.

The numerical temporal step was set to 26.7 ns and 54 ns for the simulations with and without the NHP skull, respectively, leading to a Courant-Friedrichs-Lewy number of 0.2 in both cases.

The H-231 FUS single-element transducer (Sonic Concepts, Bothell, WA) was then modeled in k-Wave. A bowl was modeled with dimensions which were comparable to the actual transducer (f_0_ = 250 kHz, outer diameter (OD) = 110 mm, inner diameter (ID) = 44 mm, and focal distance = 110 mm). The geometric focus was placed 3 cm below the skull surface. The skull was rotated using imrotate3 MATLAB function such that it created different incidence angles. The pixel resolution was 0.4 × 0.4 × 0.4 mm with a 3D grid composed of total of 271 × 215 × 215 voxels. The CT data was resampled to fit the k-Wave simulation voxel size. The maximum pressure was recorded for every voxel in the simulation grid. A total of 8 simulations were performed at 8 different incidence angles. Additionally, another simulation was performed to mimic free-field. Resulting pressure fields were used to obtain values of focal shift, focus full width at half maximum (FWHM), and skull insertion loss. The relationship between attenuation and incidence angle was then established. For each incidence angle simulation case, the maximum focal pressure value (p_skull_), used for the attenuation calculation, was semi-automatically calculated within a cube-shape volumetric ROI (region of interest) where it was expected to be generated, within the volume that corresponds to the location where the brain would be located in a realistic scenario. Then, for the attenuation calculation, the focal maximum pressure value p_skull_ was compared to the one (p_FF_) generated by the single-element transducer focusing in free-field in the absence of the skull (i.e., in water). The attenuation (a_skull_), in percentage relative to the focal pressure in free-field p_FF_, was finally calculated following the expression: a_skull_ (%) = 100(p_FF_ − p_skull_)/p_FF_. The 3D incidence angle of the FUS beam onto the sonicated skull was calculated between the central axis of the transducer (propagation direction) and a plane fitted to the sonicated skull surface (Fig. 3c). We first obtained the grid points corresponding to the sonicated skull surface by evaluating the size of the focusing cone of the beam, generated by the single-element transducer, over the skull. Thereafter, we fitted the resulting surface to a plane. Finally, we calculated the incidence angle from the normal vector of this plane as 90° − α, where α is the auxiliary angle between the normal vector of the skull fitted plane and the propagation direction vector of the transducer.

### Cranial harmonic B-mode imaging

To obtain 3D transcranial skull map, B-mode images of two ex-vivo skulls were acquired at 2MHz with phased array (P4-2, ATL, Philips) using using 256 tilted diverging waves ranging from −30 degrees to 30 degrees (Vantage 256, Verasonics). The sub-aperture used for excitation was 20.48 mm^50^. B-mode images were acquired at a depth of 110 mm. To obtain a single B-Mode image, a 2-cycle diverging wave at 2MHz followed by another 2-cycle diverging wave with opposite polarity was delivered to perform harmonic imaging. After acquisition of a B-Mode image of a plane, imaging transducer was moved 0.2 mm to acquire image of the next plane. A total of 500 planes were acquired to reconstruct image of an entire skull. This process was repeated for the second skull. After obtaining B-Mode image of each plane, skull was segmented out using ‘roipoly’ function of MATLAB (Fig. 3b). Following skull segmentation across all planes, a 3D skull map was reconstructed. After raster scan, B-Mode slices of skull was compared to skull CT of skull number 1 to identify similar slices. Once identified, skull thickness from CT was compared to skull thickness obtained from B-Mode slices (Fig. 4c and d). Skull thickness for skull number 1 was computed for 3 different slices. For skull number 2, calipers were used to measure distances at 4 different positions on the physical skull (Fig. 5c). The measurements from calipers were compared to those made based on B-Mode. Our initial 3D scan was aimed at reconstructing the whole skull map.

### Ex-vivo setup for transcranial pressure-field distribution measurement, attenuation, incidence angle, and skull thickness assessment

In order to compute incidence angles for FUS procedure, we acquired only 70 slices, 0.5 mm apart using a robotic arm (Universal Robots, UR5E), which was used to cover 35 mm of the NHP ex-vivo skull. The imaging transducer and the FUS transducer (F0 = 250 kHz, OD = 110 mm, ID = 44 mm, and focal distance = 110 mm), coaxially aligned with the imaging one, were moved together. FUS pulses of 15 cycles at a PRF of 100 Hz at 0.3 MPa were transmitted thereafter. The resulting pressure fields were recorded using a bullet hydrophone (Onda) (Fig. 3a). A free field recording was also performed in absence of the skull and was compared to pressure fields obtained in the presence of skull. An average of 4 raster scans in 2D were performed sequentially alternating axial and transversal planes until finding the maximum pressure value generated transcranially, starting with the hydrophone centered at the approximated acoustic focal maximum pressure coordinate as indicated by the manufacturer’s datasheet. Then, a transverse 2D raster scan was performed across the focus, the maximum pressure coordinate was identified, and the hydrophone was positioned at that coordinate as reference position for the next measurement plane. Next, an axial 2D raster scan was performed, the maximum pressure coordinate was identified, and the hydrophone was positioned at that coordinate as reference. This sequential 2-plane method was repeated until finding the absolute maximum pressure value within the focal volume generated transcranially. The attenuation was calculated following the expression described in this “Methods and Materials” section (sub-section “Simulations using k-Wave”). In order to evaluate different incidence angles, the skull was manually rotated. A total of 10 pressure fields, including one in free field, was obtained. After obtaining the pressure field and NHP ex-vivo B-Mode images, the images were segmented as previously mentioned. After segmenting the images, the incidence angles were calculated following the method described in this “Methods and Materials” section (sub-section “Simulations using k-Wave”). Lastly, for the thickness evaluation to compare with the ground-truth CT map of the skull, rigid body FSL registration^51^ was performed, allowing a precise comparison at the same skull region. Given the different nature of both skull imaging techniques, before the registration step, we found convergence into a successful result by converting to binary masks both CT and B-Mode skull maps. The threshold used for this binarization was of 400 HU (all values below were set to zero since they belong to soft tissues) for the CT map, and 0.19 (normalized amplitude) for the B-Mode.

### In-vivo harmonic B-Mode imaging feasibility

To show the feasibility of transcranial imaging in NHPs, we performed in-vivo imaging in two NHPs (Male rhesus-macaques, 8 years old). All procedures were reviewed and approved by the Columbia University Institutional Animal Care and Use Committee and performed in accordance with the relevant guidelines and regulations for animal research. Additionally, our study followed the ARRIVE guidelines. In the first NHP, we imaged coronal slices. For our second NHP, we imaged sagittal slices. We mounted our neuronavigation^30,37,38^ system onto the robotic arm . Once mounted onto the robotic arm, 2 cycle diverging waves at 2 MHz followed by 2 cycle diverging waves at 2 MHz with opposite polarity were delivered to acquire harmonic images of the skull. A total of 256 tilted diverging waves ranging from −30 degrees to 30 degrees were used. A total of 4 frames were acquired, saved, and then robotic arm was moved to another plane 0.5 mm away from the previous plane. This procedure was repeated until 500 planes of data were acquired for each NHP. The planes were then segmented off-line (Fig. 6b) and were assembled to generate a 3D skull map of the in-vivo NHPs.

### Supplementary Information

Supplementary Figure S1.

## Data Availability

The dataset generated and used in this study will be available from the corresponding author upon request.

## References

[1] Deuschl, G., Schade-Brittinger, C., Krack, P., Volkmann, J., Schäfer, H., Bötzel, K., Daniels, C. et al. *A Randomized Trial of Deep-Brain Stimulation for Parkinson’s Disease A BS TR AC T.* Retrieved from www.nejm.org (2006). 10.1056/NEJMoa060281 16943402

[2] Fenoy AJ, Simpson RKJ (2014). Risks of common complications in deep brain stimulation surgery: Management and avoidance. J. Neurosurg..

[3] Joo EY, Han SJ, Chung S-H, Cho J-W, Seo DW, Hong SB (2007). Antiepileptic effects of low-frequency repetitive transcranial magnetic stimulation by different stimulation durations and locations. Clin. Neurophysiol..

[4] Airan R (2017). Neuromodulation with nanoparticles. Science.

[5] Woods AJ, Antal A, Bikson M, Boggio PS, Brunoni AR, Celnik P, Cohen LG (2016). A technical guide to tDCS, and related non-invasive brain stimulation tools. Clin. Neurophysiol..

[6] Fishman PS, Elias WJ, Ghanouni P, Gwinn R, Lipsman N, Schwartz M, Chang JW (2018). Neurological adverse event profile of magnetic resonance imaging-guided focused ultrasound thalamotomy for essential tremor. Mov. Disord..

[7] Lipsman N, Schwartz ML, Huang Y, Lee L, Sankar T, Chapman M, Hynynen K (2013). MR-guided focused ultrasound thalamotomy for essential tremor: A proof-of-concept study. Lancet Neurol..

[8] di Biase L, Falato E, Caminiti ML, Pecoraro PM, Narducci F, di Lazzaro V (2021). Focused ultrasound (FUS) for chronic pain management: Approved and potential applications. Neurol. Res. Int..

[9] Abrahao A, Meng Y, Llinas M, Huang Y, Hamani C, Mainprize T, Aubert I (2019). First-in-human trial of blood–brain barrier opening in amyotrophic lateral sclerosis using MR-guided focused ultrasound. Nat. Commun..

[10] Mainprize T, Lipsman N, Huang Y, Meng Y, Bethune A, Ironside S, Heyn C (2019). Blood-brain barrier opening in primary brain tumors with non-invasive MR-guided focused ultrasound: A clinical safety and feasibility study. Sci. Rep..

[11] Chang JW, Park CK, Lipsman N, Schwartz ML, Ghanouni P, Henderson JM, Gwinn R (2018). A prospective trial of magnetic resonance-guided focused ultrasound thalamotomy for essential tremor: Results at the 2-year follow-up. Ann. Neurol..

[12] Voogt MJ, Trillaud H, Kim YS, Mali WPThM, Barkhausen J, Bartels LW, Deckers R (2012). Volumetric feedback ablation of uterine fibroids using magnetic resonance-guided high intensity focused ultrasound therapy. Eur. Radiol..

[13] Karakatsani MEM, Samiotaki GM, Downs ME, Ferrera VP, Konofagou EE (2017). Targeting effects on the volume of the focused ultrasound-induced blood-brain barrier opening in nonhuman primates in vivo. IEEE Trans. Ultrason. Ferroelectr. Freq. Control.

[14] Karakatsani, M. E., Samiotaki, G., Downs, M., Ferrera, V., and Konofagou, E. Targeting effects on the volume of the focused-ultrasound-induced blood-brain barrier opening in Non-Human Primates in vivo. In *2015 IEEE International Ultrasonics Symposium, IUS 2015*. doi:10.1109/ULTSYM.2015.0071 (2015). 10.1109/TUFFC.2017.2681695 PMC5542068 28320656

[15] Wu SY, Sanchez CS, Samiotaki G, Buch A, Ferrera VP, Konofagou EE (2016). Characterizing focused-ultrasound mediated drug delivery to the heterogeneous primate brain in vivo with acoustic monitoring. Sci. Rep..

[16] Tung Y-S, Choi JJ, Baseri B, Konofagou EE (2010). Identifying the inertial cavitation threshold and skull effects in a vessel phantom using focused ultrasound and microbubbles. Ultrasound Med. Biol..

[17] Tung Y-S, Vlachos F, Choi JJ, Deffieux T, Selert K, Konofagou EE (2010). In vivo transcranial cavitation threshold detection during ultrasound-induced blood-brain barrier opening in mice. Phys. Med. Biol..

[18] Choi JJ, Pernot M, Small SA, Konofagou EE (2007). Noninvasive, transcranial and localized opening of the blood-brain barrier using focused ultrasound in mice. Ultrasound Med. Biol..

[19] Choi JJ, Wang S, Tung Y-S, Morrison B, Konofagou EE (2010). Molecules of various pharmacologically-relevant sizes can cross the ultrasound-induced blood-brain barrier opening in vivo. Ultrasound Med. Biol..

[20] Errico C, Pierre J, Pezet S, Desailly Y, Lenkei Z, Couture O, Tanter M (2015). Ultrafast ultrasound localization microscopy for deep super-resolution vascular imaging. Nature.

[21] Heiles B, Chavignon A, Hingot V, Lopez P, Teston E, Couture O (2022). Performance benchmarking of microbubble-localization algorithms for ultrasound localization microscopy. Nat. Biomed. Eng..

[22] Mozaffarzadeh, M., Verweij, M. D., Daeichin, V., De Jong, N., and Renaud, G. Transcranial Ultrasound Imaging with Estimating the Geometry, Position and Wave-Speed of Temporal Bone. In *2021 IEEE International Ultrasonics Symposium (IUS)* pp. 1–4. doi:10.1109/IUS52206.2021.9593826 (2021).

[23] Bertolo A, Nouhoum M, Cazzanelli S, Ferrier J, Mariani JC, Kliewer A, Belliard B (2021). Whole-brain 3D activation and functional connectivity mapping in mice using transcranial functional ultrasound imaging. J. Visual. Exp..

[24] MacÉ E, Montaldo G, Cohen I, Baulac M, Fink M, Tanter M (2011). Functional ultrasound imaging of the brain. Nat. Methods.

[25] Demene C, Baranger J, Bernal M, Delanoe C, Auvin S, Biran V, Alison M (2017). Functional ultrasound imaging of brain activity in human newborns. Sci. Transl. Med..

[26] Singh A, Kusunose J, Phipps MA, Wang F, Chen LM, Caskey CF (2022). Guiding and monitoring focused ultrasound mediated blood–brain barrier opening in rats using power Doppler imaging and passive acoustic mapping. Sci. Rep..

[27] Lee SA, Kamimura HAS, Burgess MT, Konofagou EE (2020). Displacement imaging for focused ultrasound peripheral nerve neuromodulation. IEEE Trans. Med. Imaging.

[28] Lee, S. A., Kamimura, H. A. S., Burgess, M. T., Pouliopoulos, A., and Konofagou, E. E. Real-Time Displacement and Cavitation Imaging of Non-Invasive Neuromodulation of the Peripheral Nervous System via Focused Ultrasound. In *2018 IEEE International Ultrasonics Symposium (IUS)* pp. 1–4. doi:10.1109/ULTSYM.2018.8580011 (2018).

[29] Lea-Banks H, O’Reilly MA, Hamani C, Hynynen K (2020). Localized anesthesia of a specific brain region using ultrasound-responsive barbiturate nanodroplets. Theranostics.

[30] Bae S, Pouliopoulos A, Ji R, Liu K, Jiménez-Gambín S, Yousefian O, Kokossis D (2023). Transcranial cavitation mapping of blood–brain barrier opening regions in Alzheimer’s disease patients using a neuronavigation-guided focused ultrasound system. J. Acoust. Soc. Am..

[31] Konofagou E (2023). Neuronavigated focused ultrasound for clinical bbb opening in Alzheimer’s and brain cancer patients. J. Acoust. Soc. Am..

[32] Konofagou E (2023). Real-time transcranial mapping in non-human primates and human subjects during opening of the blood-brain barrier. J. Acoust. Soc. Am..

[33] Paff M, Boutet A, Boutet A, Neudorfer C, Elias GJB, Germann J, Loh A (2020). Magnetic Resonance-Guided Focused Ultrasound Thalamotomy to Treat Essential Tremor in Nonagenarians. Stereotact Funct Neurosurg.

[34] H. Zhou, Y. Liu, X. Long, Y. Qiao, J. Lee, X. Liu, H. Zheng, et al., MR-guided blood-brain barrier opening induced by rapid short-pulse ultrasound in non-human primates. Retrieved from https://qims.amegroups.com/article/view/64169. 10.21037/qims-20-1047 PMC8107301 34079712

[35] Jones RM, O’Reilly MA, Hynynen K (2015). Experimental demonstration of passive acoustic imaging in the human skull cavity using CT-based aberration corrections. Med. Phys..

[36] Liu H, Sigona MK, Manuel TJ, Chen LM, Caskey CF, Dawant BM (2022). Synthetic CT skull generation for transcranial MR imaging–guided focused ultrasound interventions with conditional adversarial networks. Proc. SPIE.

[37] Pouliopoulos AN, Wu S-Y, Burgess MT, Karakatsani ME, Kamimura HAS, Konofagou EE (2020). A clinical system for non-invasive blood-brain barrier opening using a neuronavigation-guided single-element focused ultrasound transducer. Ultrasound Med. Biol..

[38] Pouliopoulos AN, Kwon N, Jensen G, Meaney A, Niimi Y, Burgess MT, Ji R (2021). Safety evaluation of a clinical focused ultrasound system for neuronavigation guided blood-brain barrier opening in non-human primates. Sci. Rep..

[39] Wu SY, Aurup C, Sanchez CS, Grondin J, Zheng W, Kamimura H, Ferrera VP (2018). Efficient blood-brain barrier opening in primates with neuronavigation-guided ultrasound and real-time acoustic mapping. Sci. Rep..

[40] Treeby BE, Cox BT (2010). Modeling power law absorption and dispersion for acoustic propagation using the fractional Laplacian. J Acoust Soc Am.

[41] Treeby BE, Jaros J, Rendell AP, Cox BT (2012). Modeling nonlinear ultrasound propagation in heterogeneous media with power law absorption using a k-space pseudospectral method. J. Acoust. Soc. Am..

[42] Jiménez N, Camarena F, Redondo J, Sánchez-Morcillo V, Hou Y, Konofagou EE (2016). Time-domain simulation of ultrasound propagation in a tissue-like medium based on the resolution of the nonlinear acoustic constitutive relations. Acta Acustica United Acustica.

[43] Tabei M, Mast TD, Waag RC (2002). A k-space method for coupled first-order acoustic propagation equations. J. Acoust. Soc. Am..

[44] Mast TD (2000). Empirical relationships between acoustic parameters in human soft tissues. Acoust. Res. Lett. Online.

[45] Schneider U, Pedroni E, Lomax A (1996). The calibration of CT Hounsfield units for radiotherapy treatment planning. Phys Med Biol.

[46] Aubry J-F, Tanter M, Pernot M, Thomas J-L, Fink M (2003). Experimental demonstration of noninvasive transskull adaptive focusing based on prior computed tomography scans. J. Acoust. Soc. Am. doi.

[47] Schoen S, Arvanitis CD (2020). Heterogeneous angular spectrum method for trans-skull imaging and focusing. IEEE Trans. Med. Imaging.

[48] Jiménez-Gambín S, Jiménez N, Benlloch JM, Camarena F (2019). Holograms to focus arbitrary ultrasonic fields through the skull. Phys. Rev. Appl..

[49] Jiménez-Gambín S, Jiménez N, Pouliopoulos AN, Benlloch JM, Konofagou EE, Camarena F (2022). Acoustic holograms for bilateral blood-brain barrier opening in a mouse model. IEEE Trans. Biomed. Eng..

[50] Grondin J, Sayseng V, Konofagou EE (2017). Cardiac strain imaging with coherent compounding of diverging waves HHS public access. IEEE Trans. Ultrason. Ferroelectr. Freq. Control.

[51] Woolrich MW, Jbabdi S, Patenaude B, Chappell M, Makni S, Behrens T, Beckmann C (2009). Bayesian analysis of neuroimaging data in FSL. NeuroImage.

