# Supplementary material for: An all-ultrasound cranial imaging method to establish the relationship between cranial FUS incidence angle and transcranial attenuation in non-human primates in 3D
Source: Sci Rep. 2024 Jan 17;14:1488. doi: 10.1038/s41598-024-51623-5 (PMC10794232; doi:10.1038/s41598-024-51623-5)
Supplement: Supplementary file 1 — Supplementary Figure S1. [file 41598_2024_51623_MOESM1_ESM.pptx]

## Slide 1
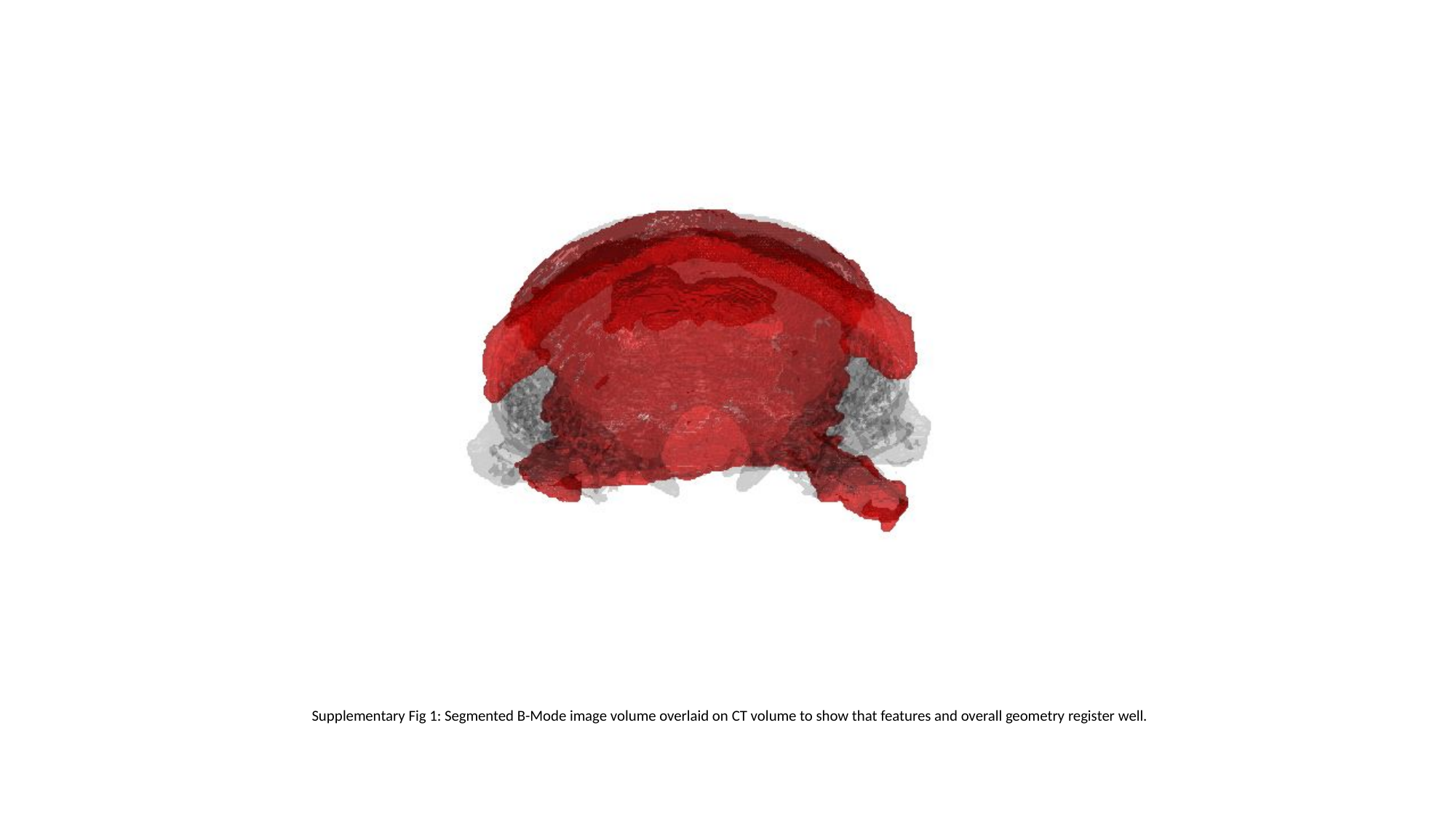

Supplementary Fig 1: Segmented B-Mode image volume overlaid on CT volume to show that features and overall geometry register well.
